# Impact of take-home messages written into slide presentations delivered during lectures on the retention of messages and the residents’ knowledge: a randomized controlled study

**DOI:** 10.1186/s12909-020-02092-7

**Published:** 2020-06-03

**Authors:** Alexandre Lautrette, Alexandre Boyer, Didier Gruson, Laurent Argaud, Carole Schwebel, Bernard Tardy, Philippe Vignon, Bruno Megarbane, Pierre Schoeffler, Pascal Chabrot, Jeannot Schmidt, Yves Boirie, Claude Guerin, Michaël Darmon, Kada Klouche, Bertrand Souweine, Jean Dellamonica, Bruno Pereira, for the TREX group, Jean-François Timsit, Nicolas Terzi, Laurent Papazian, Marc Gainnier, Antoine Roch, Jean-Marie Forel, Sami Hraiech, Nathanaël Eisenmann, Julien Bohe, Jean-Christophe Richard, Martin Cour, Fabrice Zeni, Guillaume Thiery, Sophie Perinel, Gilles Bernardin, Boris Jung, Olivier Jonquet, Stein Silva

**Affiliations:** 1 Intensive Care Medicine, Gabriel Montpied Hospital, University Hospital of Clermont-Ferrand, Clermont-Ferrand, France; 2 Intensive Care Unit, Centre Jean Perrin, Clermont-Ferrand, France; 3 LMGE «Laboratoire Micro-organismes: Génome et Environnement», UMR CNRS 6023, Clermont-Auvergne University, Clermont-Ferrand, France; 4 Intensive Care Unit, Pellegrin-Tripode Hospital, University Hospital of Bordeaux, Bordeaux, France; 5 Intensive Care Unit, Edouard Herriot Hospital, University Hospital of Lyon, Lyon, France; 6 Intensive Care Unit, Albert Michallon Hospital, University Hospital of Grenoble, Grenoble, France; 7 Clinical Investigation Center-CIC 1408, Nord Teaching Hospital, University Hospital of Saint-Etienne, Saint-Etienne, France; 8 Medical-Surgical Intensive Care Unit, Dupuytren Hospital, University Hospital of Limoges, Limoges, France; 9 Department of Medical and Toxicological Critical Care, Lariboisière Hospital, Assistance Publique - Hopitaux de Paris, INSERM UMRS-1144, Paris-Diderot University, Paris, France; 10 Intensive Care Unit, Department of Anaesthesiology, Gabriel Montpied Hospital, University Hospital of Clermont-Ferrand, Clermont-Ferrand, France; 11 Department of Radiology, Gabriel Montpied Hospital, University Hospital of Clermont-Ferrand, Clermont-Ferrand, France; 12 Adult Emergency Department, Gabriel Montpied Hospital, University Hospital of Clermont-Ferrand, Clermont-Ferrand, France; 13 Nutrition Unit, Gabriel Montpied Hospital, University Hospital of Clermont-Ferrand, Clermont-Ferrand, France; 14 Intensive Care Unit, Croix Rousse Hospital, University Hospital of Lyon, Lyon, France; 15 Intensive Care Unit, Nord Teaching Hospital, University Hospital of Saint-Etienne, Saint-Etienne, France; 16 Intensive Care Unit, Lapeyronie Hospital, University Hospital of Montpellier, Montpellier, France; 17 Medical Intensive Care Unit, Gabriel Montpied Hospital, University Hospital of Clermont-Ferrand, Clermont-Ferrand, France; 18 Intensive Care Unit, l’Archet Hospital, Cote d’Azur University, Nice, France; 19 Biostatistics unit, Delegation à la Recherche Clinique (DRCI), University Hospital of Clermont-Ferrand, Clermont-Ferrand, France

**Keywords:** Medical education, Resident, Lecture, Knowledge, Take-home message

## Abstract

**Background:**

Lectures with slide presentations are widely used to teach evidence-based medicine to large groups. Take-home messages (THMs) are poorly identified and recollected by students. We investigated whether an instruction to list THMs in written form on slides would improve the retention thereof by residents, and the residents’ level of knowledge, 1 month after lectures.

**Methods:**

Prospective blinded randomized controlled study was conducted. Twelve lectures (6 control and 6 intervention lectures) were delivered to 73 residents. For the intervention lectures, the lecturers were instructed to incorporate clear written THMs into their slide presentations. The outcomes were ability of resident to recollect THMs delivered during a lecture (as assessed by accordance rate between the lecturers’ and residents’ THMs) and knowledge (as assessed by multiple choice questions (MCQs)).

**Results:**

Data for 3738 residents’ THMs and 3410 MCQs were analyzed. The intervention did not significantly increase the number of THMs written on slides (77% (*n* = 20/26), 95% CI 56–91 vs 64% (*n* = 18/28), 95% CI 44–81, *p* = 0.31) nor THMs retention (13% (*n* = 238/1791), 95% CI 12–15 vs 17% (*n* = 326/1947), 95% 15–18, *p* = 0.40) nor knowledge (63.8 ± 26.2 vs 61.1 ± 31.4 /100 points, *p* = 0.75). In multivariable analyses performed with all THMs written on slides from the two groups, a superior knowledge was associated with notetaking during lectures (OR 1.88, 95% CI 1.41–2.51) and THMs retention (OR 2.17, 95% CI 1.54–3.04); and THMs retention was associated with written THMs (OR 2.94, 95% CI 2.20–3.93).

**Conclusions:**

In lectures delivered to residents, a third of the THMs were not in written form. An intervention based on an explicit instruction to lecturers to provide THMs in written form in their slide presentations did not result in increased use of written THMs into the slide presentation or improvement of the THMs retention or level of knowledge. However, we showed that there was a strong positive association between writing THMs on a slide, retention of THMs and residents’ knowledge. Further researches are needed to assess interventions to increase written THMs in lectures by faculty.

**Trial registration:**

ClinicalTrials.gov NCT01795651 (Fev 21, 2013).

## Background

Medical education is fundamental to improve the quality of health care but also poses great challenges [1]. Several approaches, such as the diversification of learning methods, have been explored to improve the quality of medical education. The emergence of simulation or problem-based learning has improved skill and competency training and had a positive impact on clinical practice [2]. These educational techniques are applied in small-groups settings to obtain immediate and personalized feedback [3, 4]. Another way to improve medical education is to enhance the effectiveness of teaching strategies [5]. Conventional methods, which are widely used to teach evidence-based medicine (EBM), are being revolutionized by the incorporation of modern communication modalities into textbooks, such as videos (Quick Response (QR) code) and audio files, and by videoclips inserted into slides for lecture presentations, and video-recorded e-learning lectures [6, 7]. Although students appreciate the wide availability of video-based learning, face-to-face education continues to be relevant and offers a contextualized educational approach [8]. In this approach, illustrative examples are provided, tailored to the audience; this promotes involvement of students and is relevant to EBM [8, 9]. Several studies have reported that face-to-face lectures are as effective as e-learning using video-recorded lectures for teaching EBM [10, 11]. However, as with all educational approaches, the efficacy of these methods is poor [10]. Currently, almost all face-to-face lectures and a large proportion of e-learning approaches, rely on slide presentations [12, 13] to highlight take-home messages (THMs). We previously reported a major failure to identify THMs by Intensive Care Unit (ICU) residents during face-to-face lectures based on slide presentations [14]. Two-thirds of ICU residents identified, at best, only one of the three main THMs at the end of lecture. We postulated that THMs in written form on slides could improve this retention because the identification of THM would be easier and the lecturer would take time to stress the mind on the message to remember. In this study, we investigated whether instructing teachers clearly to write THMs on their slide presentations would improve the rate of retention thereof by residents, and residents’ knowledge assessed 1 month after a critical care lecture.

## Methods

### Study setting and participants

We conducted a prospective randomized controlled blinded study at the University of Clermont-Ferrand, France, in February 2013 during the delivery of an ICU educational module (part of a postgraduate Intensive Care Unit (ICU) diploma program) attended by 25% of the national residents enrolled on the ICU training course. We enrolled all residents and lecturers who took part in the module into this study, except those who refused to participate. Written informed consent was obtained retrospectively from lecturers and prospectively from residents. The study protocol was approved by the Ethics Committee of the French Intensive Care Society (CE SRLF, No.12–394) and the local Institutional Review Board (IRB00008526, No.201837) in accordance with French law. The study was registered on the Clinical Trials Registry (clinicaltrials.gov) NCT01795651.

### Study design and intervention

The educational module was delivered over 5 consecutive days during which 12 expert lecturers each gave a single lecture pertaining to EBM for critical illness (Additional file 1). The residents were instructed to attend all lectures and they were blinded to the intervention. They were sent the lecture topics 1 month before the module and therefore had the opportunity to prepare questions in advance. Interaction between the audience and lecturers was actively encouraged. All lecturers were experienced teachers who attended faculty workshops on effective lecture presentations, including how to devise an educational slide presentation and communicate THMs.

Two months before the educational module, the lecturers were randomly assigned in a 1:1 ratio to the intervention or control lectures group, by permuted-block randomization (i.e. random block sizes) using a computer-generated random allocation (Stata software). They were blinded to the study and their group assignment during the preparation and delivery of their lecture. The title and educational objectives of the lecture were chosen by the lecturer**.** The invitation e-mail provided information on the lecture conditions (face-to-face lecture with a slide presentation), the duration (30 min followed by 15 min for questions) and the learners’ characteristics (postgraduate resident doctors enrolled on an ICU training programme). At 1 month and at 1 week before the beginning of the module, a reminder e-mail was sent to each lecturer. The intervention element of the study was an explicit instruction to lecturers to include at least one slide entitled “Message” or “Take-home Message” into their slide presentations containing the written THM, for each THM delivered. A THM was defined as a short message of key relevant to medical practice. The number of THMs was limited to five per lecture. The choice and the wording of the THMs were decided on the lecturer. Each written THM was limited to 15 words. The instruction to the lecturers was clearly stated in a separate paragraph within the e-mail, which also included an article reporting the failure of residents to identify THMs in ICU postgraduate lectures [14]. The email did not mention that the lecture to be delivered was part of an experimental study. In the final part of the e-mails, the lecturers were encouraged to contact us for further details in the event of any problems or misunderstanding. The lecturers in the control group received only the three invitation e-mails, while those in the intervention group received the three e-mails including the instructional paragraph.

The lecturers were later informed about the study at the end of their lecture. All accepted to take part in the study. After the lecture, the lecturers provided the investigators with up to five THMs (≤ 15 words per THM) that they had included in their lecture, and a maximum of five multiple choice questions (MCQs) with answers related to their THMs. The lecturers in the two groups indicated if their THMs had been explicitly written or not in the slide presentation. For all 12 lectures, two blinded teacher-reviewers compared the THMs written on slides in the slide presentation of the lecturer with the report of THMs written or not on slides given by lecturer after the lecture. There was no disagreement between lecturers and reviewers. This assessment validated the THMs written or not on slides in all lectures.

The residents were informed about the study and gave consent to participate on the first day of the module, before the first lecture. However, they were blinded to the precise nature of the intervention, and to the outcomes, although they knew that they would be contacted 1 month after the final lecture to assess their knowledge of the module’s content. This assessment was not associated with other stake for the residents. One month after the last lecture, the residents completed an assessment form sent by e-mail. For each lecture, they were asked if they had attended the lecture, the THMs that they recollect (≤ 15 words per THM) and whether they had taken notes (defined as writing down the key points of the lecture). The definition of a THM was provided in the e-mail. The number of THMs delivered by the lecturer during each lecture was specified in assessment form. The residents did not have access to slide presentations but could consult their notes or learning materials when answering the assessment. They were allowed to give one additional THM to the number of THMs delivered during the lecture to increase the opportunity to recollect the THMs delivered by lecturer. Finally, they answered MCQs related to the different THMs. For each MCQ, the residents were given the number of correct answers (Additional file 2). Two reminder e-mails were sent to residents who did not reply; if there was still no reply, they were secondarily excluded (Additional file 3).

### Outcomes

The primary outcome was the difference in THMs retention as assessed by the rate of accordance between the THMs delivered by lecturers and given by residents, between the intervention and control lectures. The accordance was independently determined by two reviewers who were intensivist teachers but not lecturers on the educational module. They were also blinded to two group assignments. A binary scoring system was used: “Yes” when there was clearly a match between the resident and lecturer messages, and “No” to all other cases. If there was disagreement between the reviewers after a second analysis of THM accordance, a third reviewer analysed the data (disagreement arose in 4.6%, of the evaluation, *n* = 174/3738). The order in which the THMs were listed on the responses forms was not taken into account in the analysis.

The second outcomes were 1) the difference in residents’ level of knowledge, as assessed by the MCQ, between the intervention and control lectures, 2) the identification of factors associated with better THMs retention or knowledge.

Each MCQ was rated 0 if there was at least one error among resident answers or 1 if there was no error. The knowledge of a resident was assessed for each lecture with a score based on the MCQs related to the lecture (total possible score, 100 points). Three groups of residents’ level of knowledge were established according to relevance and statistical distribution (interquartile range): low performance (< 50 points), medium performance (50–80 points) and high performance (> 80 points). If a resident failed to attend a lecture, no score was recorded.

### Statistical analysis

At least 70 residents would participate in the educational module. Assuming that they would all attend all 12 lectures, 420 assessment forms were expected per lecture groups. In our previous observational study, the THM accordance rate observed was 39% at the end of lecture [14]. In the present study, the primary endpoint was assessed 1 month after the last lecture. Therefore, we assumed a 50% relative decrease in accordance, such that the expected THM accordance rate was 20%. For a two-sided type I error at 5%, 3500 residents’ THMs (i.e. 1750 THMs per lecture group) would have a power of 80% to show an absolute difference of 5% (20% vs. 25%), taking into account between- and within- resident’s variability measured using intra-cluster correlation coefficient.

All statistical analyses were performed with Stata statistical software (version 13, StataCorp, College Station, US). Categorical data are expressed as numbers and percentages, and quantitative parameters as mean ± standard-deviation (SD) or median [interquartile range], according to statistical distribution. The normality of the data was assessed using the Shapiro-Wilk’s test. To take into account variability between and within lecturers and residents, random-effect models were generated (lecturers and residents as crossed random effects). These models (generalized linear mixed model with logit link function) were used to determine factors associated with THMs retention and residents’ knowledge. Multivariable analyses were then performed, with covariates (fixed effects) determined according to their significance in univariate analysis (*P* < 0.10) and clinical relevance, for THMs retention: gender, slides per lecture, notetaking and THMs written on slides; for residents’ knowledge: gender, notetaking and THMs retention. Particular attention was paid to multicollinearity and the interactions between covariates, and the impact of adding variables to, or omitting them from, the multivariable model. Results were expressed as odds-ratios (OR) or adjusted odds-ratios (aOR) for multivariable analyses and 95% confidence intervals (95% CI). Sensitivity analysis was conducted with THMs retention rate treated as a continuous variable (using negative binomial generalized linear mixed model) and categorized according to various cut-offs, such as a cut-off of 25% determined according to the expected assumption used for sample size estimation.

## Results

**Fig. 1:**
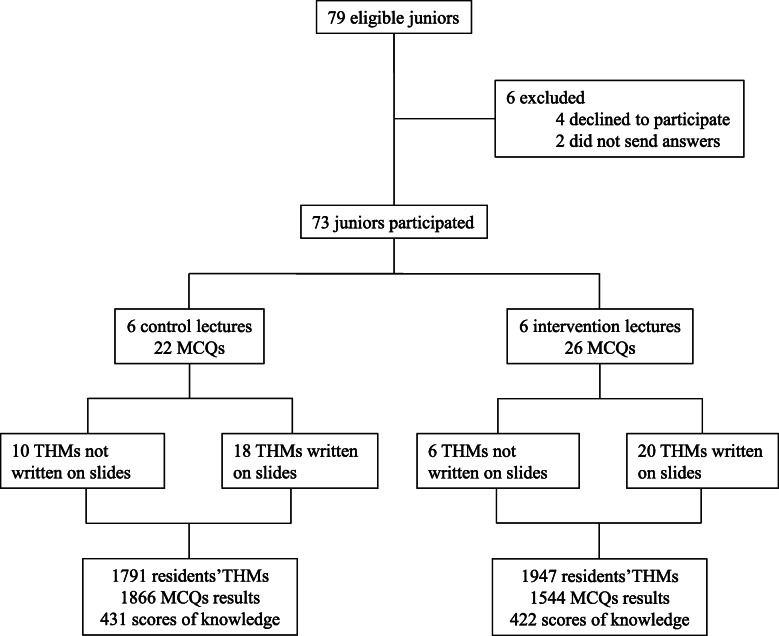
Flow chart of study. Legend: THM, take-home message

Of the 79 eligible residents who attended the educational module, 4 declined to participate and 2 did not return the questionnaire and were then secondarily excluded (Fig. 1). The data from 73 residents were finally analyzed. The residents population was predominantly male (*n* = 50, 68%) with an age of 31 ± 3 years and 2 ± 1 years of ICU experience. The two lecturer groups were similar at baseline in terms of general characteristics and lecture characteristics (Table 1). Each of the 12 lectures was attended by a mean of 71 residents, achieving the analysis of 853 scores, 3738 residents’ THMs and 3410 MCQs.

**Table 1:** Characteristics of lectures and lecturers

Variable	Lecturers *n* = 12	Control group *n* = 6	Intervention group *n* = 6
Male, n (%)	11 (92)	6 (100)	5 (83)
Teaching experience (years), mean ± SD	22 ± 10	23 ± 10	21 ± 11
Professor of medicine, n(%)	11 (92)	5 (83)	6 (100)
Lecture delivered in the afternoon, n(%)	6 (50)	2 (33)	4 (67)
Slides per lecture (number), mean ± SD	53 ± 12	50 ± 9	56 ± 15
THMs per lecture (number), mean ± SD	4.5 ± 0.8	4.7 ± 0.5	4.3 ± 1.0
Written THMs per lecture (number), mean ± SD	3.2 ± 1.4	3.0 ± 1.4	3.3 ± 1.5
MCQs per lecture (number), mean ± SD	4.0 ± 0.9	4.3 ± 0.8	3.7 ± 0.8

*THM* take-home message, *MCQ* multiple choice question

### Assessment of THMs retention

**Fig. 2:**
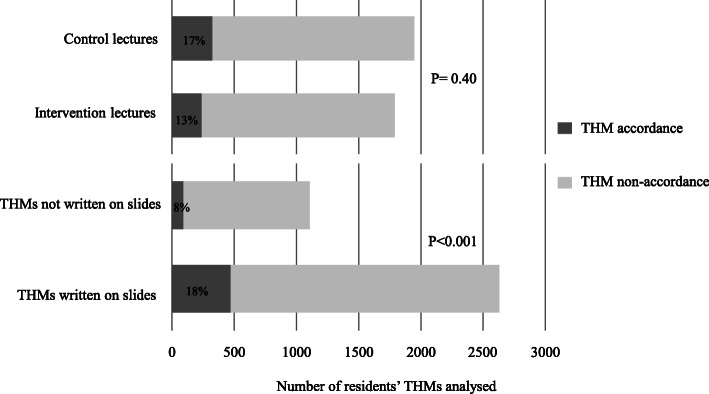
Retention of THMs by residents. Legend: Dark gray bars correspond to the number of accordances between the lecturer’s THMs and the resident’s THMs; Gray bars correspond to the number of non-accordances between the lecturer’s THMs and the resident’s THMs. THM, take-home message

The number of written THMs incorporated into the slide presentations did not differ between the intervention and control groups (77% (*n* = 20/26), 95% CI 56–91 vs 64% (*n* = 18/28), 95% CI 44–81, *P* = 0.31). The rate of THMs retention was not different between the intervention and control groups (13% (*n* = 238/1791), 95% CI 12–15 vs 17% (*n* = 326/1947), 95% CI 15–18, *P* = 0.40) (Fig. 2).

### Assessment of residents’ knowledge

Residents’ knowledge was assessed by 22 MCQs related to the THMs in the intervention lectures, and by 26 MCQs in the control lectures. The level of knowledge in the intervention lectures did not have higher than that in the control lecture (63.8 ± 26.2 vs 61.1 ± 31.4 points, *P* = 0.75).

### Impact of written THMs on a slide on THMs retention

Of the 54 THMs delivered during the 12 lectures, 38 (70%) appeared in written form on a slide. The THMs retention was higher for written THMs compared to THMs delivered only orally (18% (*n* = 473/2630), 95% CI 17–20 vs 8% (*n* = 91/1108), 95% CI 7–10, *P* < 0.001) (Fig. 2). In univariate (Table 2) and multivariable analysis adjusted for randomization lectures group and the number of slides, the factors associated with THMs retention were the writing of THMs on slides (aOR = 2.94, 95% CI 2.20–3.93; *P* < 0.001) and notetaking by resident during the lecture (aOR = 2.05, 95% CI 1.70–2.48; *P* < 0.001).

**Table 2:** Univariate analysis of THMs retention

Variable	THM non-accordance *n* = 3174	THM accordance *n* = 564	OR [95% CI], *P* value
Intervention lectures, n (%)	1553 (48.9)	238 (42.2)	0.79 [0.45–1.38], *p* = 0.40
Male, n (%)	2202 (69.4)	381 (67.6)	0.92 [0.76–1.11], *p* = 0.39
THMs written on slides, n (%)	2157 (68.0)	473 (83.9)	2.99 [2.24–3.99], *p* < 0.001
Notetaking, n (%)	1297 (40.9)	341 (60.5)	2.04 [1.69–2.47], *p* < 0.001
Slides per lecture, (number)	57 [46–60]	53 [44–58]	0.97 [0.95–0.99], *p* = 0.008
Lecture in afternoon, n (%)	1489 (46.9)	242 (42.9)	0.92 [0.52–1.65], *p* = 0.79
Teaching experience (years)	21.4 ± 8.8	22.6 ± 9.3	1.02 [0.99–1.05], *p* = 0.30

*THM* take-home message

### Impact of THMs retention on residents’ knowledge

The relationship between THMs retention and residents’ knowledge was significant (*P* < 0.001): the THMs retention was 10.9 ± 16.1%, 13.0 ± 18.5% and 22.3 ± 25.2% in the low-, medium-, and high-performance groups, respectively. Univariate analyses comparing knowledge, gender, notetaking (yes/no) and THMs retention (≤ or > 25%) between the control and intervention lectures groups are shown in Table 3. In the multivariable analysis adjusted for randomization group and gender, better knowledge was associated with notetaking (aOR = 1.88, 95% CI 1.41–2.51; *p* < 0.001) and a higher rate of THMs retention (aOR = 2.17, 95% CI 1.54–3.04; *p* < 0.001). Sensitivity analysis with THMs retention as continuous variable (aOR = 1.02, 95% CI 1.01–1.03, *p* < 0.001), and then with THMs retention thresholds of 20% (aOR = 1.69, 95% CI 1.26–2.26, *p* < 0.001) and 30% (aOR = 2.24, 95% CI 1.56–3.22, *p* < 0.001), was conducted to verify that this choice of THMs retention threshold did not affect the relationship between THMs retention and knowledge.

**Table 3:** Univariate analysis of residents’ knowledge

	Performance groups (score out of 100 points) *n* = 853				
**Variable**	Mean ± SD (score/100 points)	Low performance (< 50 points) *n* = 250 (29%)	Medium performance (50–80 points) *n* = 359 (42%)	High performance (> 80 points) *n* = 244 (29%)	*P* value
Lectures					
Control	63.8 ± 26.2	117 (27.2)	185 (42.9)	129 (29.9)	0.75
Intervention	61.1 ± 31.4	133 (31.5)	174 (41.2)	115 (27.3)	
Gender of resident					
Female	64.4 ± 29.3	75 (28.1)	102 (38.2)	90 (33.7)	0.04
Male	61.6 ± 28.7	175 (29.9)	257 (43.9)	154 (26.3)	
Notetaking					
No	58.5 ± 28.9	159 (32.6)	219 (44.9)	110 (22.5)	< 0.001
Yes	67.9 ± 28.0	91 (24.7)	140 (38.5)	134 (36.8)	
THMs retention					
≤25%	58.2 ± 29.0	217 (33.9)	279 (43.6)	144 (22.5)	< 0.001
> 25%	75.2 ± 24.7	33 (15.5)	80 (37.6)	100 (47.0)	

*THM*, take-home message

## Discussion

In this study, an intervention based on explicit instructions to lecturers to provide THMs in written form in their slide presentations did not result in increased use of written THMs into the slide presentation or improvement of the THMs retention or level of knowledge, 1 month after a lecture. However, the multivariable analyses performed with all THMs written on slides, whatever the group, showed that the writing of THMs on slides increased THMs retention that was strongly associated with better residents’ knowledge.

The intervention assessed in our study aimed to increase the number of THMs written on slides. But the lecturers in the intervention group who were instructed to provide written THMs did not significantly change their educational practice during the study, such that there was no difference between the two lectures groups in rate of THMs retention or level of knowledge. There are several possible explanations. Firstly, the instructions provided to the intervention group may have been insufficiently clear, although the request was easy to implement for teachers with experience in giving slide presentations. We encouraged the lecturers to contact us if they encountered any problems or required further information. None did so. Secondly, we cannot rule out the possibility that the lecturers in the intervention group were unwilling to modify the format of their presentation. They may have thought that the THMs delivered during their lecture were already effectively communicated during their slide presentation. However, our results point to the opposite conclusion. Although the intervention was ineffective, the data from multivariate analyses performed with all written THMs from both groups, support the original hypothesis that written THMs are valuable. Findings from learning studies suggest that messages are more easily identified when written as a short passage of text that can be readily assimilated by the learner [15, 16]. The writing of short THMs on a slide makes notetaking easier for trainees [17]. The notetaking from written THMs contain key terminology related to the topic, which is of great help for understanding and recollecting the lecture content. The transfer of THMs is achieved orally by listening to the lecture and visually by looking at the slide presentation [17]. We instructed the lecturers to use a specific THM format but did not make it mandatory, which could have led to a significant, but artificial, difference. Changing methods or practices is highly challenging, despite convincing data published in major journals regarding the benefits thereof [18]. Implementing such changes is a long-term process that requires commitment. Targeted training for new teachers, followed by continuing education regarding new findings pertaining to the science of learning, could convince teachers to update their pedagogical approach and thus improve educational practice.

Our study had some limitations. Firstly, we did not assess the clinical impact of the knowledge acquired. However, many studies have reported a positive impact of knowledge on clinical practice [1] and none have provided any evidence to the contrary. Secondly, we did not investigate the influence of the lecture topic or the choice of lecturer on our learning outcomes. Thirdly, we did not assess the association between written THMs and residents ‘knowledge because the MCQs of lecturers were not standardized according to the level of difficulty. Fourthly, we cannot rule out that the MCQs could help the residents to remember the THMs because the MCQs and THMs asked the residents were on the same form. Fifthly, the design of the study, in which the lecturer was blinded, prevented us from assessing the quality of the THMs and the intervention. Some lecturers who were randomly assigned to the control group had standard teaching practice for putting written THMs into the slide presentation when some lecturers in the intervention group unfortunately did not follow the instructions and did not put a written THM into their slide presentation. We did not want to change the real practice of some control lecturers so as not to cause a disadvantage for the control group. If the THMs in the intervention lectures have been modified before the lecture (by adding them to slide, and potentially removing them from the slide presentations delivered in the control lectures), the control lectures would have been at a disadvantage. In addition, modification of THMs by investigators who are not experts is difficult, because THMs written on slides are an integral part of a lecturer’s oral presentation and cannot be standardized without introducing a high degree of artificiality. Thus, we retained real teaching conditions and sought to determine the effect of being instructed to include written THMs in presentations only on experienced teachers. In this real-life teaching situation, one third of the THMs delivered by the lecturers were not provided in written form on their slides.

## Conclusions

This study showed that in lectures delivered to residents, a third of the THMs did not appear in written form on the lecturers’ slides. An intervention based on an explicit instruction to lecturers to incorporate written THMs into their slide presentation did not result in increased use of written THMs into the slide presentation or improvement of the THMs retention or level of knowledge. However, the analyses performed with all THMs written on slides from the two groups of this randomized controlled study showed that there were strong positive associations between writing THMs on slides, THMs retention and knowledge of residents. Further researches are needed to assess interventions to increase written THMs in lectures by faculty.

## Supplementary information

**Additional file 1.** List of lectures.

**Additional file 2.** Examples of Multiple Choice Questions.

**Additional file 3.** Timing of the study (Figure). Legend: THM, take-home message; MCQ: multiple choice question.

## Data Availability

The data that support the findings of this study are available from the corresponding author upon reasonable request.

## Abbreviations

aOR: adjusted Odds-Ratio
CI: Confidence Intervals
EBM: Evidence-Based Medicine
ICU: Intensive Care Unit
MCQ: Multiple Choice Question
OR: Odds-Ratio
SD: Standard-Deviation
THM: Take-Home Message
