# Supplementary material for: Impact of take-home messages written into slide presentations delivered during lectures on the retention of messages and the residents’ knowledge: a randomized controlled study
Source: BMC Med Educ. 2020 Jun 3;20:180. doi: 10.1186/s12909-020-02092-7 (PMC7271544; doi:10.1186/s12909-020-02092-7)
Supplement: Supplementary file 1 — Additional file 1. List of lectures. [file 12909_2020_2092_MOESM1_ESM.docx]

**Additional file 1**

**List of lectures:**

Intervention group:

- Guidelines for drug abuse
- Safety and clinical risk management in the ICU
- Muscle metabolism in critically ill patients
- Analgesia guidelines for trauma
- Management of thrombotic microangiopathy in the ICU
- Heat stroke management

Control group:

- Cardio-renal syndrome management
- Pulmonary embolism guidelines
- Acute kidney Injury: definition and biomarkers
- Guidelines for intra-abdominal trauma
- Antithrombotic treatment and bleeding management
- Management of major trauma
