# Supplementary material for: Impact of take-home messages written into slide presentations delivered during lectures on the retention of messages and the residents’ knowledge: a randomized controlled study
Source: BMC Med Educ. 2020 Jun 3;20:180. doi: 10.1186/s12909-020-02092-7 (PMC7271544; doi:10.1186/s12909-020-02092-7)
Supplement: Supplementary file 2 — Additional file 2. Examples of Multiple Choice Questions. [file 12909_2020_2092_MOESM2_ESM.docx]

**Additional file 2**

**Examples of Multiple Choice Questions**

1) What are the diagnostic elements of thrombotic microangiopathy? (3 correct answers)

A: hemolytic anemia with thrombocytopenia

B: mechanical anemia

C: renal failure

D: neurological symptoms

E: elimination of other diagnosis

2) What are the elements of the trauma triad of death? (3 correct answers)

A: metabolic acidosis

B: hypoxemia

C: low blood pressure

D: hypothermia

E: coagulopathy

3) What is the correct answer about prehospital analgesia in trauma? (1 correct answer)

A: the regional anesthesia is the gold standard treatment

B: non-steroidal anti-inflammatory drugs are needed

C: opioid analgesics are the gold standard treatment

D: the use of opioid analgesics is only via the subcutaneous injection

4) What are the correct answers about the heat stroke? (2 correct answers)

A: the gold standard treatment is composed by paracetamol and dantrolene

B: the cooling is the gold standard treatment

C: the symptomatic treatment of organ failures is the main element of the critical care

D: the objective of the cooling is the normalization of body temperature in one hour

E: the surface cooling methods are always used

5) What are the causes of the anabolic resistance in a shock? (4 correct answers)

A: insulin resistance

B: splanchnic sequestration of amino acid

C: lipotoxicity

D: increased blood flow in the muscle

E: inflammation
