# Supplementary material for: Impact of take-home messages written into slide presentations delivered during lectures on the retention of messages and the residents’ knowledge: a randomized controlled study
Source: BMC Med Educ. 2020 Jun 3;20:180. doi: 10.1186/s12909-020-02092-7 (PMC7271544; doi:10.1186/s12909-020-02092-7)
Supplement: Supplementary file 3 — Additional file 3. Timing of the study (Figure). Legend: THM, take-home message; MCQ: multiple choice question. [file 12909_2020_2092_MOESM3_ESM.docx]

**Additional file 3**

**Figure: Timing of the study**

February

5 days: educational module

At the end of lecture, each lecturer was informed of the study and provided the investigators with :

- THMs
- MCQs

6 intervention lectures with slide presentation

Control group of lecturers:

3 Invitation e-mails

Intervention group of lecturers:

3 Invitation e-mails

+

Explicite instruction

March

E-mail to residents: assessment form

- To recollect THMs

- To answer MCQs

Lecturers’ THM

Residents’THM

3 blinded reviewers:

Assessment of THMs accordance

6 control lectures with slide presentation

December-January

Period when all lecturers are blinded to the study
